# Ambient Air Pollution Exposures and Newly Diagnosed Pulmonary Tuberculosis in Jinan, China: A Time Series Study

**DOI:** 10.1038/s41598-018-35411-6

**Published:** 2018-11-27

**Authors:** Yao Liu, LiangLiang Cui, LuJian Hou, ChunBao Yu, NingNing Tao, JinYue Liu, YiFan Li, ChengChao Zhou, GuoRu Yang, HuaiChen Li

**Affiliations:** 1 Department of Respiratory Medicine, Shandong Provincial Hospital Affiliated to Shandong University, Jinan, Shandong China; 2 Department of Biostatistics, School of Public Health, Shandong University, Jinan, Shandong, China; Jinan Municipal Center for Disease Control and Prevention, Jinan, Shandong China; 3 Jinan Research Academy of Environmental Sciences, Jinan, Shandong China; 4 Shandong Chest Hospital, Jinan, Shandong China; 5 School of Public Health, Key Lab of Health Economics and Policy Research, Shandong University, Jinan, Shandong China; 6 Department of Respiratory Medicine, Weifang No.2 People’s Hospital, Weifang, Shandong China

## Abstract

Few epidemiological studies have evaluated the effects of air pollution on the risk of pulmonary tuberculosis (TB). We investigated the associations of ambient air pollutants (particulate matter with aerodynamic diameter <2.5 μm (PM_2.5_), sulfur dioxide (SO_2_),nitrogen dioxide (NO_2_), ozone (O_3_), and carbon monoxide (CO)) in relation to the risk of pulmonary TB in a cohort of Chinese TB patient in Jinan city from 2011 to 2015. A total of 9344 newly diagnosed pulmonary TB cases were included. Poisson regression model was employed to estimate the risk of air pollution and daily diagnosed pulmonary TB. Four different air pollution exposure windows (3, 6, 9, and 12 months) before TB diagnoses were calculated from the daily concentration of air pollution. In overall analysis, we did not find strong evidence for an association between continuous exposures to most ambient air pollutants and risk for pulmonary TB. However, in categorical analysis, we observed statistically significant overall associations between pulmonary TB risk and PM_2.5_ (3 month exposure window: RR = 1.228, 95%CI: 1.091–1.381) as well as CO (3 month exposure window: RR = 1.169, 95%CI: 1.028–1.329; 9 month exposure window: RR = 1.442, 95%CI: 1.028–2.024) exposures. Moreover, subgroup analyses suggested that most of the air pollutants (PM_2.5_, SO_2_, O_3_, and CO) were significantly associated with increased risk of TB among the males, the females, the <60 years, and the smear negative cases. The dominant statistically significant associations were detected at 3-month exposure window of air pollution before the diagnosis of TB. Our results detected positive associations between ambient PM_2.5_, CO exposures and the risk of newly diagnosed pulmonary TB in China. The suggestive evidence that the 3 month air pollution exposure window was associated with increased TB risk warrants further investigation.

## Introduction

Although relatively efficacious treatment for pulmonary tuberculosis (TB) has existed for decades, the disease continues to remain one of the leading causes of mortality attributed to an infectious disease^1^. Globally, pulmonary TB causes an estimated 2 million deaths per year, the majority of which occur in the developing world^2^. Over the past 60 years, many studies have demonstrated that the risk of pulmonary TB was associated with tobacco smoking^3^. A number of studies also demonstrated that passive smoking and indoor air pollution from biomass fuels are risk factors for TB^4,5^. The above observations invite speculating about the possible role of outdoor urban air pollution, which is a growing problem in developing countries where the burden of disease from TB is also great^6^. Despite this, relatively few large-scale epidemiologic analyses have explored the association of outdoor air pollution and the risk of pulmonary TB.

Recently, the health effects of severe air pollution have attracted increasing concern in China, partly due to the increasing number of days with very high levels of air pollution. Since 2011, northern China has experienced frequent severe smog episodes, particularly in winter^7^. High concentrations of particulate matters substantially reduce visibility by absorbing and scattering sunlight and lead to detrimental effects on human health^8^. Subsequent epidemiologic studies have provided evidence of the effects of gaseous pollutants and PM on cardiovascular disease and respiratory disease^9–12^. However, little attention has been paid in Chinese studies to the assessment of the health impact of outdoor air pollutants on TB. Few epidemiologic studies conducted in developed countries have examined the potential roles of ambient air pollutants in the risk of TB, and results have been mixed. A recent nested case-control study showed that monoxide (CO) and nitrogen dioxide (NO_2_) are associated with increased risk of pulmonary TB among residents in Northern California, but no positive associations between PM with aerodynamic diameter <2.5 μm (PM_2.5_) or ozone (O_3_) and TB were observed^13^. Another U.S.-based analysis suggested a significant correlation of smear-positive TB cases and residential exposure to PM_2.5_^14^. A South Korea study reported that ambient sulfur dioxide (SO_2_) but not O_3_, CO, or NO_2_ increased the risk of TB in males^15^. A recent retrospective Chinese study suggested that long-term exposure to PM_2.5_ may increase the risk of death from TB and other diseases among TB patients^16^. Nevertheless, evidence for the relationship between outdoor air pollution and the risk of TB is limited in developing countries with a high incidence of TB and high exposure to outdoor air pollution.

We further examined these questions using a large database of 9344 resident TB cases from the National Infectious Disease Surveillance system in Jinan, China, a highly polluted large urban city over a 5-year period (from 2011 to 2015). We conducted a time-series study to investigate whether exposure to ambient air pollutants PM_2.5_, SO_2_, NO_2_, O_3_, and CO, are associated with increased risk of newly diagnosed pulmonary TB. In addition, we further examined the priority of different air pollution exposure windows before pulmonary TB diagnosis on the associations between air pollutants and TB.

## Materials and Methods

### Ethics

Ethics approval was obtained from the Ethics Committee of Shandong Provincial Hospital, affiliated with Shandong University, Shandong, China. The Ethics Committee waived the need for informed consent due to the anonymity of the data collected and the retrospective nature of this study.

### Setting

**Figure 1:**
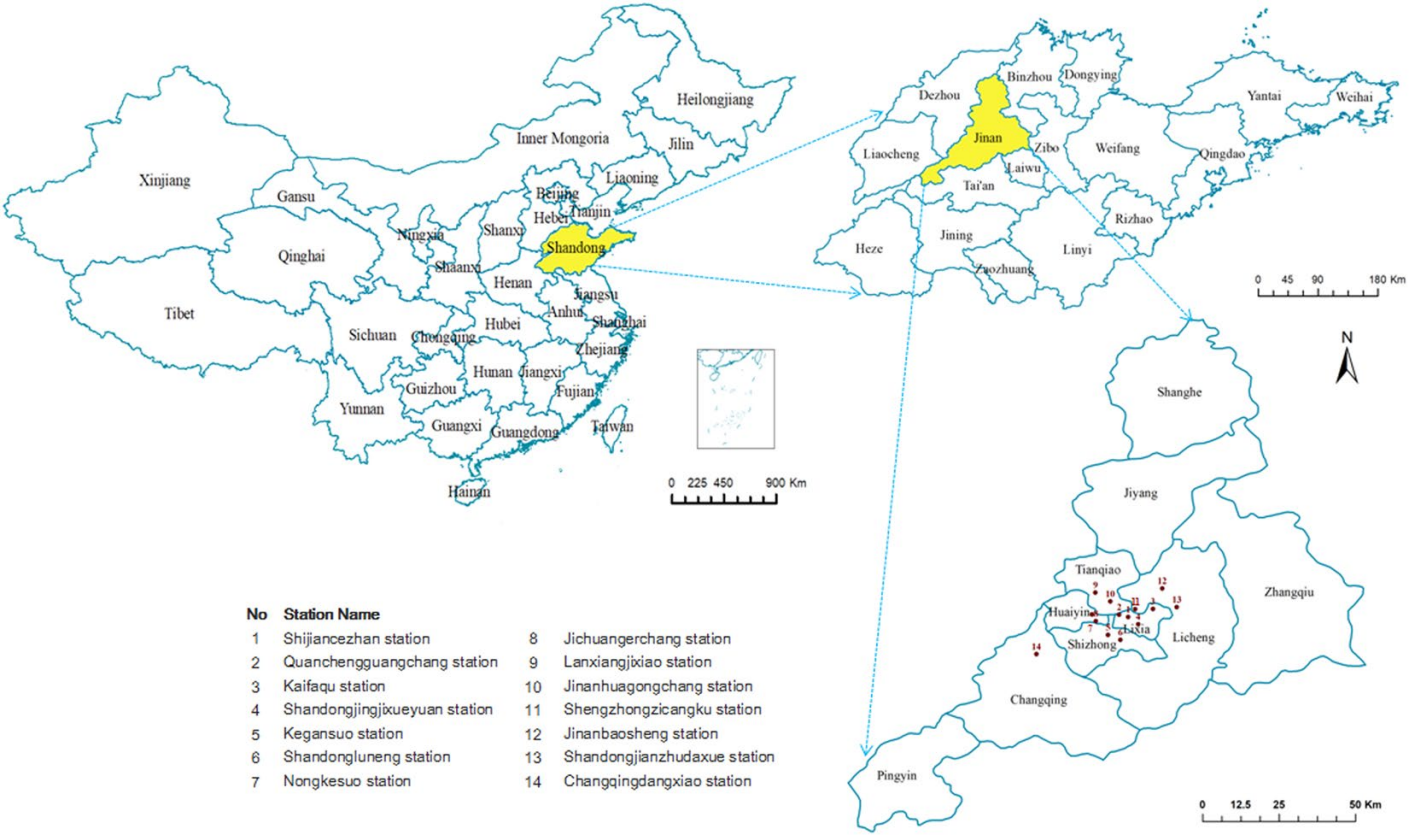
Map of Jinan city and the air quality monitoring stations.

This study was carried out in Jinan (Fig. 1), a large urban city in the eastern part of China, with a resident population of approximately 700,000 inhabitants. It is located at 36.40°N latitude and 110.00°E longitude and is 8,227 square kilometers. Jinan has a warm temperate continental monsoon climate with clear seasons, hot summers and temperate winters. The special small basin terrain makes the air fluidity is poor, and the dust easily accumulates in the sky of the city. Jinan has rapidly industrialized in the past few decades and added millions of vehicles to its highways, and the urban centeris characterized by heavy traffic congestion, particularly in rush hours.

### Study Population

Information on new TB cases in Jinan from 1 January 2011 to 31 December 2015, were obtained from the Jinan Municipal Center for Disease Control and Prevention (JMCDC), which has access to the China National Notifiable Disease Surveillance System. TB cases in the JMCDC database included all newly diagnosed active pulmonary TB cases where either the disease was proven by isolation of Mycobacterium tuberculosis or, in the absence of bacteriological confirmation, disease diagnosis based on clinical, radiologic, and/or histologic grounds together with prescription for at least two anti-tuberculosis medications including isoniazid, rifampin, ethambutol, and pyrazinamide. For TB cases, data on age, gender, home address, onset of index symptom, first hospital visiting date,diagnosis date, comorbidity, use of anti-TB drugs and relevant laboratory assays were extracted from the JMCDC clinical data bases.

### Air pollution data

Daily mean hourly air pollutant concentration data (inhalable PM_2.5_, SO_2_, NO_2_, and CO) and daily maximum of 8-hourly running mean of O_3_ were obtained from the Environmental Monitoring Center of Jinan from January 1, 2011 to December 31, 2015.The data were obtained from 14 fixed monitoring stations (Fig. 1).The pollutant monitoring network spanned the entire region, including 11 sites located in urban areas and 3 sites located in a suburban county. Daily average values of air pollution were used in this study and calculated from the above 14 fixed monitoring stations.

If 4 or more hours of air pollutant in one day in each monitoring station were missing, it was defined as a missing day for this pollutant in this station. Three or more monitoring stations were not available for the air pollutant in one day were regarded as daily missing value for the city. At least 75% daily concentration for each pollutant in each station was regarded as valuable monitoring for the year estimation. The missing daily concentration was calculated as the daily average value of the air pollutant in other stations. Otherwise, the moving average value was estimated asthe day before and after. The daily missing rate of each air pollutant for 14 monitoring stations was less than 15% (0.00% to 14.82%) in each year from 2011 to 2015, respectively (Supplementary Table S1).

### Meteorological data

We collected daily weather information for the period from January 1, 2011 to December 31, 2015 from the China Meteorological Science Data Sharing Service Network (http://cdc.cma.gov.cn/home.do). We downloaded the following data: daily average temperature and daily average relative humidity.

### Exposure window definition

Different air pollution exposure windows were defined as follows: 3-month exposure window refers to 3 months’ air pollution exposure before the diagnosis of pulmonary TB. The concentration of the 3-month exposure window was calculated by the mean of daily air pollution within the 90 days before the diagnosis of TB. Accordingly, the 6-month exposure window, 9-month exposure window and 12-month exposure window were for 6 months (180 days), 9months (270 days) and 12 months (360 days) of exposure. The date of pulmonary TB diagnosis was extracted from the JMCDC database.

### Statistical analyses

Poisson regression model was used to explore the association between air pollution and the risk of daily pulmonary TB incidence. First, we explored the risk of air pollution continuous exposure and pulmonary TB incidence in different scenario of air pollution exposure windows. To control for the confounding factors of long-term time trends and seasonal patterns and meteorological parameters, the smoothing spline functions with 7 degrees of freedom per year for time and 3 degrees of freedom for the moving average of ambient temperature and relative humidity in the same exposure time periods as the air pollutants were included in the regression model.

Next, we conducted a further estimation of the risk of air pollution categorical exposure and pulmonary TB incidence in different scenario of air pollution exposure windows. We transmitted the continuous variable of air pollution concentration in different air pollution exposure windows into a 3-level classified variable, including low-level (L), middle-level (M), and high-level (H). The median and 75 percentile value of daily average concentration of each pollutant from 2011 and 2015 were used as the cutoff values. The same classification was adopted for each air pollutant for 3-, 6-, 9,- and 12-month exposure windows. L, M, and H exposure was defined as <median value of daily air pollution, median value to 75 percentile value of daily air pollution, and >75 percentile value of daily air pollution, respectively. The L was regarded as the reference level in the regression model, and the effects of M versus L and H versus L were estimated separately.

Subgroup analysis was conducted according to gender, age group,and sputum smear tests. We also performed additional sensitivity analyses to examine the effects of air pollution on TB risk in multipollutant models compared with single-pollutant model. Risk ratios (RRs) and 95% confidence intervals (CIs) associated with the risk of TB incidence for increased exposure of air pollutants was calculated. All statistical analyses were conducted using R software (version 3.3.2).

## Results

### Description of TB cases and air pollution

We identified a total of 9344 newly diagnosed cases of pulmonary TB in Jinan city during the study period from 2011 to 2015. The mean (SD) age of these patients was 45.6 (19.7) years, and 2770 (29.6%) patients were >60 years of age. A third (3114, 33.3%) of the study population were female. The baseline characteristics of the study population according to sputum smear tests are presented in Table 1. Compared with the sputum smear-negative cases, the smear-positive cases were more likely to be men, older age, and less likely to be students and farmers. The majority of the study population lived in rural area, and the percentage of urban versus rural dwellers was comparable for sputum smear-positive cases and the controls.

**Table 1:** Demographic of tuberculosis cases by smear status from 2011 to 2015, Jinan city.

	All patients (%)	Smear- negative TB (%)	Smear- positive TB (%)	p
Number	9344 (100)	6555 (100)	2789 (100)	
Age, yr	45.6 ± 19.7	44.6 ± 19.6	47.9 ± 19.8	<0.001
Age group, yr				<0.001
<20	629 (6.7)	506 (7.7)	123 (4.4)	
20–34	2834 (30.3)	2057 (31.4)	777 (27.9)	
35–49	1678 (18.0)	1184 (18.1)	494 (17.7)	
50–59	1433 (15.3)	973 (22.0)	460 (23.9)	
>60	2770 (29.6)	1835(28.0)	935 (33.5)	
Gender				<0.001
Male	6230 (66.7)	4228 (64.5)	2002 (71.8)	
Female	3114 (33.3)	2327 (35.5)	787 (28.2)	
Occupation				<0.001
Farming	5038 (53.9)	3482 (55.8)	1556 (53.1)	
Government services and commerce	335 (3.6)	231 (3.7)	104 (3.5)	
Nonagricultural labor	748 (8.0)	522 (8.0)	229 (8.2)	
Student	799 (8.6)	642 (9.8)	157 (5.6)	
House wife or unemployed	1235 (13.2)	886 (13.5)	349(12.5)	
Retired person	632 (6.7)	396 (6.0)	236 (8.4)	
Area of residence				0.244
Urban/periurban	3780 (40.5)	2677 (40.8)	1103 (39.5)	
Rural	5564 (59.5)	3878 (59.2)	1686 (60.5)	

Table 2 presents a summary of the daily air pollution concentrations and meteorological conditions in Jinan. The mean concentrations of PM_2.5_, SO_2_, NO_2_, O_3_, and CO were 100 μg/m^3^, 77 μg/m^3^, 54 μg/m^3^, 98 μg/m^3^, and 1401 μg/m^3^, respectively. Table 3 shows the Spearman correlation coefficients between air pollutants from 2011 to 2015. PM_2.5_, SO_2_, NO_2_ and CO was positively correlated with each other, whereas O_3_ had negative correlation with other pollutants in winter.

**Table 2:** Descriptive characters of daily air pollution and meteorology indicators from 2011 to 2015, Jinan city.

Item	Mean (SD)	Min	P25	Median	P75	Max
Air pollutants(ug/m^3^)						
PM_2.5_	100 (60)	16	60	86	122	443
SO_2_	77 (56)	12	40	58	97	429
NO_2_	54 (22)	13	38	50	65	165
O_3_	98 (57)	10	49	88	141	270
CO	1401 (682)	445	963	1228	1638	6555
Meteorology						
Average temperature (°C)	14.8 (10.6)	−9.4	5.0	16.5	24	34
Relative humidity (%)	56 (20)	13	40	55	70	100

**Table 3:** Spearman’s correlation between air pollutants in Jinan, China, 2011–2015. The summer months (May–September) are shown above the diagonal; the winter months (October–April) are shown below the diagonal.

	SO_2_	NO_2_	PM_2.5_	O_3_	CO
SO2		0.752*	0.360*	0.174*	0.557*
NO2	0.728*		0.428*	−0.045	0.701*
PM2.5	0.649*	0.732*		0.252*	0.599*
O3	−0.486*	−0.428*	−0.334*		−0.086
CO	0.718*	0.828*	0.876*	−0.504*	

*P < 0.05.

### The temporal relation of monthly TB cases and air pollution

**Figure 2:**
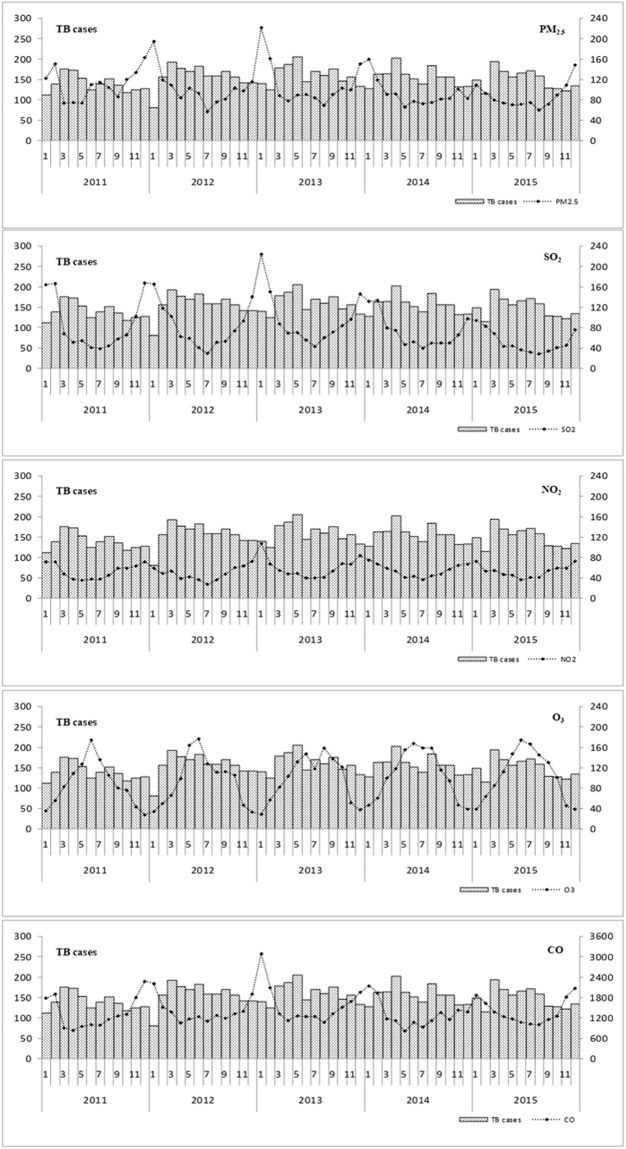
Monthly mean values of air pollutants and number of TB cases, 2011–2015.

The monthly number of TB cases exhibited a seasonal trend, with more cases in the spring months (March, April and May) than the winter months (December, January and February) (Fig. 2). The numbers of TB cases ranged from 120 to 180 per month. The overall monthly air pollution concentrations exhibited annual cyclical patterns and various temporal patterns (Fig. 2). Of the pollutants, PM_2.5_, SO_2_, NO_2_, and CO had higher concentrations observed in winter, but O_3_ had higher concentrations in summer. The overall monthly mean value of air pollution concentration in Jinan was high. Lagged correlation effects were observed between the monthly number of TB cases and pollutant concentrations, with approximately 3 to 4 months lag for PM_2.5_, SO_2_, NO_2_, and CO.

### Risks of TB incidence and air pollution

**Figure 3:**
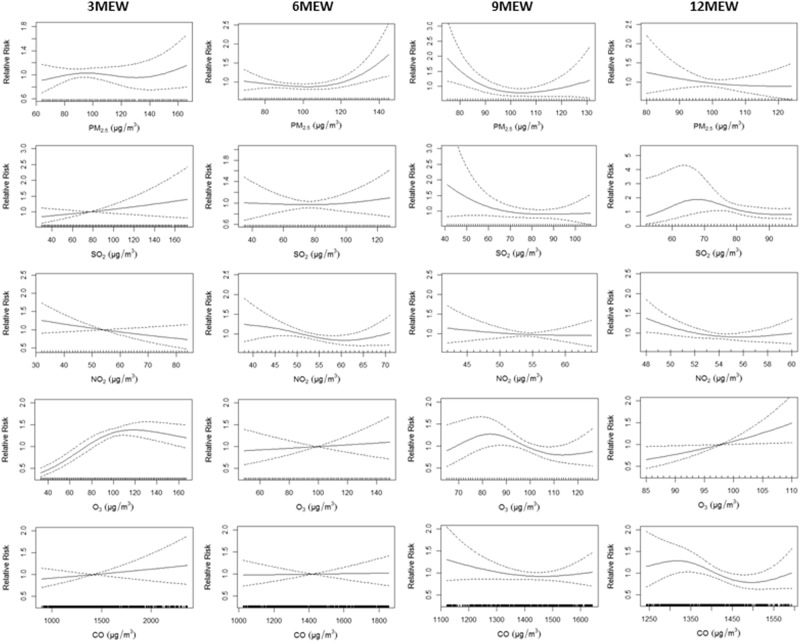
RRs and 95% CIs in risk of TB incidence with air pollutants per 10 ug/m^3^ increased at different air pollution exposure windows 3 MEW denoted three months exposure before tuberculosis diagnosis. The similar definition to the 6 MEW, 9 MEW, and 12 MEW.

The cumulative association between an increase of 10 μg/m^3^ in pollutants and the risk of newly diagnosed TB are summarized in Table 4 and graphically presented in Fig. 3. When air pollutants exposures were defined as continuous variables, weak positive associations were found for PM_2.5_ (3 month), SO_2_ (3 and 6 month), CO (3 month), and O_3_ (3,6 and 12 month), but the majority of the analyses did not reach statistical significance.The risk of newly diagnosed TB was significantly increased with O_3_ per 10 ug/m^3^ increase at the 3-month exposure window (RR = 1.076, 95% CI: 1.011–1.145) and 12-exposure window (RR = 1.389, 95% CI: 1.041–1.852).

**Table 4:** RRs and 95% CIs in the risk of daily pulmonary TB incidence associated with air pollutants per 10 ug/m^3^ increased at different air pollution exposure windows.

Pollutants	RR	95% CI
PM_2.5_		
3 month^*^	1.020	0.968, 1.074
6 month	0.963	0.875, 1.060
9 month	0.868	0.731, 1.031
12 month	0.946	0.766, 1.167
SO_2_		
3 month^*^	1.036	0.978, 1.098
6 month	1.011	0.935, 1.094
9 month	0.935	0.806, 1.084
12 month	0.843	0.653, 1.089
NO_2_		
3 month^*^	0.902	0.780, 1.042
6 month	0.776	0.604, 0.997
9 month	0.922	0.679, 1.252
12 month	0.754	0.491, 1.156
CO		
3 month^*^	1.002	0.997, 1.007
6 month	1.001	0.993, 1.008
9 month	0.994	0.980, 1.007
12 month	0.989	0.969, 1.010
O_3_		
3 month^*^	1.076	1.011, 1.145
6 month	1.020	0.937, 1.110
9 month	0.963	0.857, 1.082
12 month	1.389	1.041, 1.852

*3 month denoted three months exposure window before tuberculosis illness onset. The similar definition to the 6 month, 9 month, and 12 month.

The exposure-response relationships of air pollutants and TB exhibited different patterns for different exposure windows (Fig. 3). A consistently decreased trend was observed in the relationship of NO_2_ and newly diagnosed TB in four exposure windows. An increasing trend was observed for the air pollutants of PM_2.5_, SO_2_, O_3_ and CO and newly diagnosed TB at 3- and 6-month exposure windows. Additionally, a linear relationship was observed for the trend of O_3_ and newly diagnosed TB at the 12-month exposure window. Most of the relationships observed at the 9- and 12-month exposure window are flat or decreasing.

Table 5 presents the RRs and 95%CIs for the risk of newly diagnosed TB according to different levels of air pollutants. At the 3-month exposure window, weak positive associations were found for exposure to the middle level of PM_2.5_, SO_2_, O_3_ and CO and the high level of PM_2.5_ and O_3_, although most analyses did not reach statistical significance. Only exposure to the middle level of PM_2.5_ (RR = 1.228, 95% CI: 1.091–1.381) and CO (RR = 1.169, 95% CI: 1.028–1.329) concentration at the 3-month exposure window was significantly associated with increasing risk of newly diagnosed TB. At the 9-month exposure window, exposure to middle-level (RR = 1.276, 95% CI: 1.028–1.583) and high-level CO (RR = 1.442, 95% CI: 1.028–2.024) were both significantly associated with increased risk of TB. At 6-and 12-month exposure windows,we found no statistically significant associations between exposures to ambient pollutants and TB risk.

**Table 5:** RRs and 95% CIs in the risk of daily pulmonary TB incidence associated with air pollutants increased at different air pollution exposure windows

Pollutants (ug/m^3^)	3 month*	6 month	9 month	12 month
PM_2.5_				
L	ref	ref	ref	ref
M	1.228 (1.091, 1.381)	0.851 (0.699, 1.003)	1.030 (0.779, 1.361)	1.039 (0.775, 1.393)
H	1.004 (0.798, 1.262)	1.188 (0.911, 1.550)	1.358 (0.877, 2.105)	1.114 (0.724, 1.714)
SO_2_				
L	ref	ref	ref	ref
M	1.025 (0.880, 1.195)	1.117 (0.933, 1.337)	1.141 (0.917, 1.419)	0.807 (0.554, 1.175)
H	0.987 (0.784, 1.243)	1.041 (0.819, 1.322)	1.148 (0.848, 1.554)	NA
NO_2_				
L	ref	ref	ref	ref
M	0.918 (0.804, 1.047)	0.929 (0.809, 1.066)	1.145 (0.961, 1.363)	0.961 (0.795, 1.162)
H	0.981 (0.806, 1.194)	1.209 (0.945, 1.548)	NA	NA
O_3_				
L	ref	ref	ref	ref
M	1.099 (0.952, 1.269)	0.747 (0.484, 1.010)	0.900 (0.764 1.061)	1.123 (0.933, 1.352)
H	1.072 (0.876, 1.311)	0.757 (0.513, 1.001)	NA	NA
CO				
L	ref	ref	ref	ref
M	1.169 (1.028, 1.329)	0.915 (0.797, 1.050)	1.276 (1.028, 1.583)	NA
H	0.957 (0.793, 1.154)	0.855 (0.694, 1.054)	1.442 (1.028, 2.024)	NA

*3 month denoted three months exposure before tuberculosis diagnosis. The similar definition to the 6 month, 9 month, and 12 month.

PM_2.5_: L = ≤ 86ug/m^3^, M = (87–122) ug/m^3^, H = >123 ug/m^3^; SO2: L = ≤58 ug/m^3^, M = (59–97) ug/m^3^, H = >98 ug/m^3^; NO2: L = ≤50 ug/m^3^, M = (51–65) ug/m^3^, H = >66 ug/m^3^; O3: L = ≤88 ug/m^3^, M = (89–141) ug/m^3^, H = >142 ug/m^3^; CO: L = ≤1228 ug/m^3^, M = (1229–1638) ug/m^3^, H = >1639 ug/m^3^. NA: not available.

**Figure 4:**
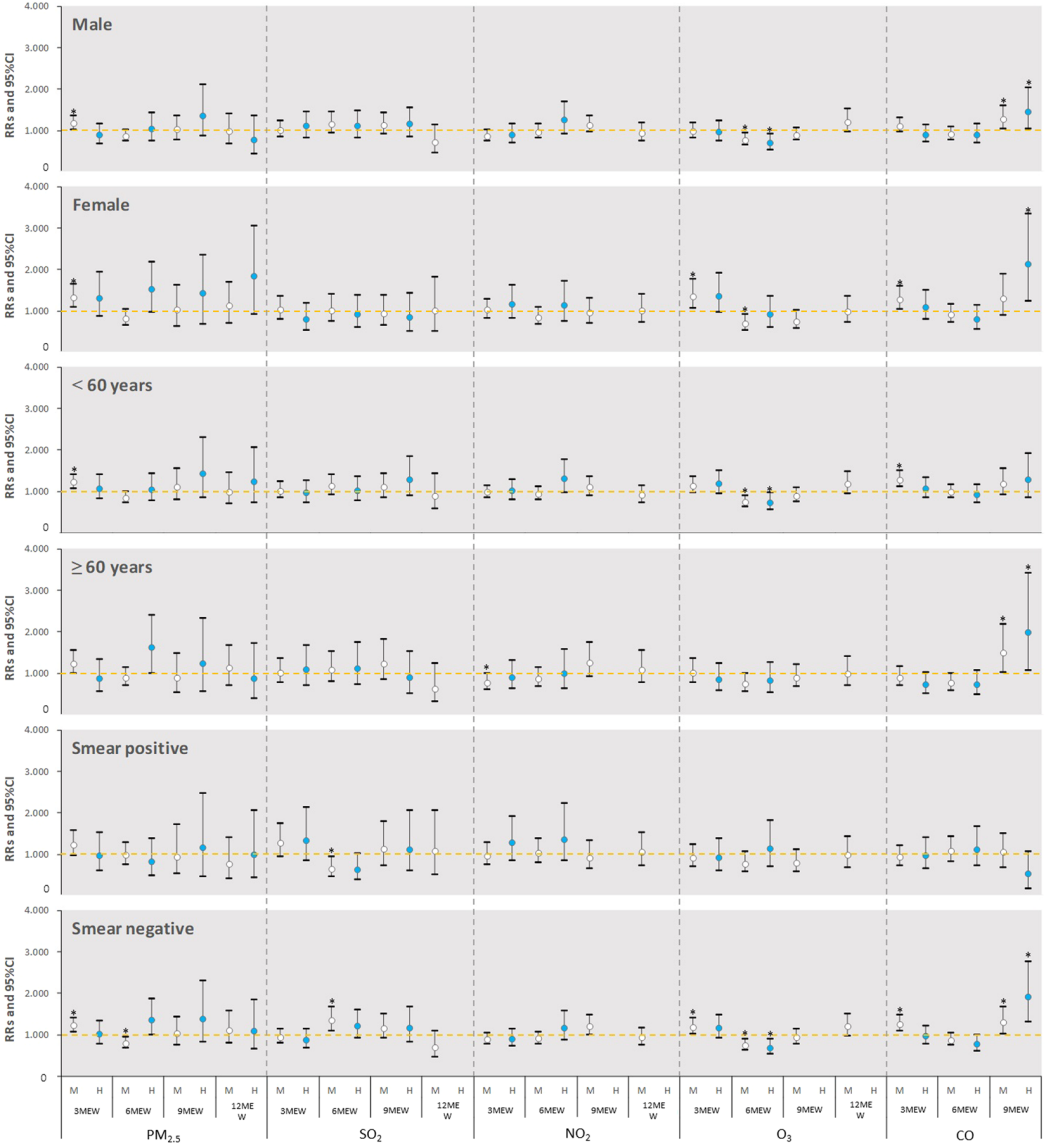
RRs and 95% CIs in risk of TB incidence and by gender, age group and smear status associated with air pollutants increased at different air pollution exposure windows 3 MEW denoted three months exposure before tuberculosis diagnosis. The similar definition to the 6 MEW, 9 MEW, and 12 MEW.

Figure 4 demonstrates that the RRs and 95% CIs for the risk of TB by gender, age group and smear status is associated with increased air pollutants at different air pollution exposure windows. For PM_2.5_, exposure to a middle level of PM_2.5_ was significantly associated with increasing risk of TB among men (RR = 1.181, 95% CI: 1.023–1.364), women (RR = 1.338, 95% CI: 1.090–1.643), <60-year-olds (RR = 1.223, 95% CI: 1.065–1.403), and the smear negative cases (RR = 1.225, 95% CI: 1.068–1.404) atthe 3-month exposure window.

For SO_2_, exposure to middle-level SO_2_ was associated with an increased risk of newly diagnosed TB among the smear negative cases (RR = 1.353, 95% CI: 1.096–1.670) but not among smear positive TB cases at the 6-month exposure window.

Exposure to O_3_ with the middle-level concentration at the 3-month exposure window was found to be associated with increasing risk of TB among women (RR = 1.364, 95% CI: 1.060–1.755) and the smear negative cases (RR = 1.195, 95% CI: 1.012–1.411). In contrast, O_3_ exposure was associated with a reduced risk of TB among women (RR = 0.696, 95% CI: 0.532–0.910), men (RR = 0.773, 95% CI: 0.639–0.935), individuals less than 60 years (RR = 0.746, 95% CI: 0.619–0.898), and smear negative patients (RR = 0.746, 95% CI: 0.622–0.895) at the 6-month exposure window.

Exposure to middle-level CO was associated with increased risk of TB among women (RR = 1.285, 95%CI: 1.030–1.285), individuals <60 years old (RR = 1.288, 95% CI: 1.109–1.497), and the smear negative patients (RR = 1.261, 95% CI: 1.086–1.466) at the 3-month exposure window. Moreover, a higher risk of TB was also observed among males (RR = 1.442, 95% CI: 1.028–2.024), females (RR = 2.116, 95% CI: 1.227–3.650), older individuals (≥60 years) (RR = 1.988, 95% CI: 1.069–3.699) and the smear negative patients (RR = 1.902, 95% CI: 1.307–2.768) for exposures to CO at the 9-month exposure window.

### Sensitivity analysis

Most of the statistically significant associations between air pollution and newly diagnosed TB were observed at the 3-month exposure window; therefore, we conducted 3 different dual-pollutant models to compare the results of the single-pollutant models at the 3-month exposure window. Table S3 presents the results of the single and dual-pollutant models. Similar results were observed in the sensitivity analyses, and the statistically significant associations between air pollution and TB risk overall and in the subgroups remained robust.

## Discussion

To the best of our knowledge, this is the first large study to assess the potential associations between short- and long-term exposure to ambient air pollutants with PM_2.5_, SO_2_, NO_2_, O_3_, or CO and the risk of newly diagnosed pulmonary TB in China. In the overall analysis, we did not find strong evidence for an association between continuous exposures to the most ambient air pollutants and risk for pulmonary TB in this sample of >7 million Chinese residents. However, when the pollutants were analyzed as categorical variables, statistically significant associations were observed between pulmonary TB risk and exposure to PM_2.5_ and CO. Moreover, subgroup analyses suggested that most air pollutants (PM_2.5_, SO_2_, O_3_, and CO) were significantly associated with increasing risk of TB among males, females, individuals <60 years old, and smear negative cases. The dominant statistically significant associations were observed at the 3-month exposure window before pulmonary TB diagnosis.

Overall, we found no strong association for ambient PM_2.5_ exposures and TB risk. Only at the 3-month exposure window, positive associations were found for exposure to high level of PM_2.5_ in the total study population and the following subgroups:males, females, individuals <60 years, and smear negative cases. Only two prior American studies investigated the effects of PM_2.5_ on the risk of TB, but the results were inconsistent^13,14^. Studies with low levels of PM_2.5_ and limited numbers of active pulmonary TB cases might lack statistical power to detect the effects of PM_2.5_. Starting from early 2011, China was hit by unprecedented air pollution, which was characterized by extremely high concentrations of ambient PM^7^. Our study was conducted in Jinan, a high polluted urban city in eastern China,with the recent 5 years of PM_2.5_ data, and the daily mean PM_2.5_ level was much higher than previous studies in China. The findings from this epidemiological study may add to the evidence for an association between exposure to higher concentrations PM_2.5_ and increased risk for pulmonary TB.

We found positive associations between CO and increased pulmonary TB risk at the 3- and 9-month exposure windows and among the subgroups including men, women, individuals <60 years old, and smear-negative cases; women exhibited the highest risk to CO at the 12-month exposure window (RR = 2.116).These results corroborate the findings of a recent Northern California population-based case-control study^13^ as well as the results of previous studies reporting that indoor air pollution caused by biomass fuel combustion increase TB risk^4,5^. CO has been identified as an intracellular signaling molecule in recent years, and the physiological effects have been found to shut down the immune response of the host^17^. CO blocks the effects of the IL-10 anti-inflammatory pathway and down regulates tumor necrosis factor-alpha (TNF-α). TNF-α plays an important role in the formation of granulomata and containment of TB^18^. Moreover, the down regulation of TNF-α also decreases the macrophage-mediated defense against TB^19^. As a result, blocking the IL-10 pathway and down regulating of TNF-α by CO in lung macrophages may promote the reactivation of TB^20^.

No significant effect of SO_2_ on TB risk was observed in the overall study population. We present evidence that ambient SO_2_ was significantly related to increased risk of pulmonary TB for the smear negative TB cases. Previous studies conducted in the US did not detect a significant effect of SO_2_ on the risk of pulmonary TB^13^. China has one of the highest SO_2_ levels in the world^21^. In 2009, the annual average SO_2_ concentration in 113 major Chinese cities was 42 ug/m^3^ ^22^, and in our study, the mean concentration of SO_2_ from 2011–2015 in Jinan was 77 ug/m^3^, all of which is higher than the reported levels in developed countries^23^. A previous time-series analysis demonstrated that ambient SO_2_ is significantly associated with all-cause and cardiopulmonary mortality in 17 Chinese cities^8^. These findings contribute to the scientific literature on the health effects of SO_2_ in China, which suggest that the Chinese government should promote “controlling/reducing total SO_2_ emissions” as a major air pollution control strategy.

Another important finding of our present study is that O_3_ contributed independently to increased risk for pulmonary TB among female TB cases (RR = 1.364) and smear negative TB cases (RR = 1.195) at the 3-month exposure window. O_3_ is now recognized as an important air pollutant that can increase health risk in China^21^. In laboratory studies, O_3_ can worsen pulmonary function, increase airway inflammation and gas exchange^24,25^. Previous epidemiological studies demonstrated that exposure to elevated concentrations of O_3_ has been associated with pulmonary dysfunction^26^ and an increased risk of respiratory mortality^27^. Interestingly, the study by Smith *et al*.^13^ observed an inverse association with O_3_ and pulmonary TB among residents in Northern California, with exposures above the lowest quintile of O_3_ resulting in decreases in pulmonary TB risk. The results were consistent with our findings that exposure to O_3_ at the6-month exposure window exhibited an inverse association with pulmonary TB. Further study elucidating the underlying mechanisms involved in the effect of O_3_ on pulmonary TB development should be pursued.

We observed no positive association between NO_2_ and pulmonary TB. We are aware of the study by Smith *et al*.^13^ reporting positive associations between NO_2_ and the risk of pulmonary TB. The heterogeneous findings may reflect different characteristics of local air pollution or patterns of exposure among residents. However, associations with NO_2_ and pulmonary TB were nonsignificant, which may indicate that this pollutant does not affect the risk of TB or that the analyses lacked statistical power to estimate small effects. Additional studies are needed to elucidate the possible associations between NO_2_ and pulmonary TB in China.

The most important strength of our study is that we described the effects of ambient air pollutants and newly diagnosed TB risk, including both continuous exposure and categorical exposure. We also examined different air pollution exposure windowsbefore TB diagnosis in the association between air pollutants and the risk of pulmonary TB. Both linear and nonlinear patterns were explored to determine the relationship between air pollutants and newly diagnosed TB across different exposure windows. In prior cohort studies, TB cases were simply assigned into a single long-term exposure, such as 24 months, and there was little evidence for each air pollutant exposure window and TB risk^13^. In the present study, we observed that monthly TB cases and air pollutant concentration were lag correlated, with significantly increased risk of TB primarily occurring after 2 to 3 months of air pollution exposure. Our estimations coincided with this correlation, as most of the statistically significant associations were observed at the 3-month exposure window. Further epidemiological studies are needed to confirm the importance of the air pollution exposure window and increased TB risk both in developed and developing countries. This information is important for determining the appropriate interventions to manage the risk of short-term and long-term air pollution exposure on health effects.

The present study has a number of limitations. First, a large proportion of TB cases were diagnosed on the basis of clinical and radiologic evidence. This might lead to some diagnosis misclassification. Our study assumed that air pollution concentrations of a fixed-monitoring site are representative of population exposure and did not account for exposure misclassification. In fact, two types of exposure misclassification were not avoidable in this study. One is a classical type, caused by variations between the ambient air pollutant level and the personal exposure level. Further, the true personal exposure varies by the outdoor level, indoor air pollution, residential characteristics, and individual time−activity patterns. This exposure misclassification tends to bias associations between air pollutants and health outcomes toward the null. The other type occurs due to spatial heterogeneity of different air pollutants levels. Ambient air pollutants were uniformly distributed within a spatial unit, but they exhibited different spatial variations at the city level. The exposure of air pollution to TB cases in our study was estimated by matching individual address to the nearest monitoring site. Therefore, some TB cases living close to the same monitor site were assumed to be exposed to the average air pollution concentration level due to limited monitoring data, not considering the spatial distribution of air pollution concentration. Additionally, most of the monitoring sites were arranged in the urban area and limited in rural area, so the exposure of TB cases in rural area was estimated less precisely. Future work should focus on obtaining a more accurate picture of exposure or new methods for assessing exposure error induced by spatial variability. For example, personal exposure variation could be estimated by a model simulation based on the concentration of fixed-monitoring sites, which would be an improved method for better representation and less exposure error. Finally, we failed to estimate the association between PM_10_ and TB cases because 2 of the 14 air pollution monitor stations had more than 25% PM_10_ measurements missed in our study period.

## Conclusions

In conclusion, although there was no statistically significant association between continuous exposure to most of the air pollutants and TB risk., we detected positive associations between ambient PM_2.5_, SO_2_, O_3_, CO and the risk of newly diagnosed TBin categorical analyses. The sensitive exposure window to air pollution was identified as the 3-month air pollution exposure beforeTB diagnosis. Our results contribute to the understanding of air pollutant exposures and the risk of pulmonary TB in China and shed some light on the difference in air pollutant effects between Eastern and Western populations.

## Electronic supplementary material

Supplementary Materials
