# Supplementary material for: Ambient Air Pollution Exposures and Newly Diagnosed Pulmonary Tuberculosis in Jinan, China: A Time Series Study
Source: Sci Rep. 2018 Nov 27;8:17411. doi: 10.1038/s41598-018-35411-6 (PMC6258663; doi:10.1038/s41598-018-35411-6)
Supplement: Supplementary file 1 — Supplementary Materials [file 41598_2018_35411_MOESM1_ESM.docx]

**Supplementary materials**

**Ambient Air Pollution Exposures and Newly Diagnosed Pulmonary Tuberculosis in Jinan, China: A Time Series Study**

Liu Yao^1#^, Cui LiangLiang^2#^, HouLuJian^3^, Yu ChunBao^4^, Tao NingNing^1^, Liu JinYue^1^, Li YiFan^1^,Zhou ChengChao^5^,Yang GuoRu^6*#^, Li HuaiChen^1*#^

Institution of authors: 1. Department of Respiratory Medicine, Shandong Provincial Hospital Affiliated to Shandong University, Jinan, Shandong, China

2. Department of Biostatistics, School of Public Health, Shandong University, Jinan, Shandong, China; Jinan Municipal Center for Disease Control and Prevention, Jinan, Shandong, China

3. Jinan Research Academy of Environmental Sciences, Jinan, Shandong, China

4. Shandong Chest Hospital, Jinan, Shandong, China

5. School of Public Health, Key Lab ofHealth Economics and Policy Research, Shandong University, Jinan, Shandong, China

6.Department of Respiratory Medicine, Weifang No.2 People’s Hospital, Weifang, Shandong, China

^#^ These authors contributed equally to this work and should be considered as co-first authors

*Corresponding author: Address correspondence to Li HuaiChen, Department of Respiratory Medicine, Shandong Provincial Hospital Affiliated to Shandong University, No.324 Jingwuweiqi Road. Jinan,250021, Shandong, China. Telephone: 0086-531-68776332. E-mail: lihuaichen@163.com

Table S1 Missing rate of daily air pollutants in each year during 2011 to 2015 in Jinan city

| Pollutant | 2011 (%) | 2012 (%) | 2013 (%) | 2014 (%) | 2015 (%) | Total |
| --- | --- | --- | --- | --- | --- | --- |
| PM_2.5_ | 14.82^*^ | 13.11^*^ | 0.80 | 1.10 | 1.00 | 6.17 |
| SO_2_ | 7.59 | 2.42 | 1.59 | 1.43 | 0.86 | 2.78 |
| NO_2_ | 7.60 | 2.42 | 1.88 | 1.29 | 0.96 | 2.83 |
| CO | 1.76 | 1.64 | 1.06 | 1.10 | 0.79 | 1.27 |
| O_3_ | 0.94 | 0.47 | 0.92 | 1.17 | 4.01 | 1.50 |

* PM_2.5_ in 2011 and 2012 was available from three monitoring stations, including Quanchengguangchang station (urban), Shandongjianzhudaxue station (urban), Lanxiangjixiao station (suburban).

Table S2 RRs and 95% CIs in the risk of daily pulmonary TB incidence associated with air pollutants per 10 ug/m^3^ increased at different air pollution exposure windows

| Pollutants | RR | 95% CI |
| --- | --- | --- |
| PM_2.5_ |  |  |
| 3 month^*^ | 1.020 | 0.968, 1.074 |
| 6 month | 0.963 | 0.875, 1.060 |
| 9 month | 0.868 | 0.731, 1.031 |
| 12 month | 0.946 | 0.766, 1.167 |
| SO_2_ |  |  |
| 3 month^*^ | 1.036 | 0.978, 1.098 |
| 6 month | 1.011 | 0.935, 1.094 |
| 9 month | 0.935 | 0.806, 1.084 |
| 12 month | 0.843 | 0.653, 1.089 |
| NO_2_ |  |  |
| 3 month^*^ | 0.902 | 0.780, 1.042 |
| 6 month | 0.776 | 0.604, 0.997 |
| 9 month | 0.922 | 0.679, 1.252 |
| 12 month | 0.754 | 0.491, 1.156 |
| CO |  |  |
| 3 month^*^ | 1.002 | 0.997, 1.007 |
| 6 month | 1.001 | 0.993, 1.008 |
| 9 month | 0.994 | 0.980, 1.007 |
| 12 month | 0.989 | 0.969, 1.010 |
| O_3_ |  |  |
| 3 month^*^ | 1.076 | 1.011, 1.145 |
| 6 month | 1.020 | 0.937, 1.110 |
| 9 month | 0.963 | 0.857, 1.082 |
| 12 month | 1.389 | 1.041, 1.852 |

^*^ 3 month denoted three months exposure window before tuberculosis illness onset. The similar definition to the 6 month, 9 month, and 12 month.

Table S3 Sensitive analysis of daily pulmonary TB incidence associated with air pollutants increased at 3 months exposure window, and stratified by gender, age group, and sputum smear status

| Pollutants (ug/m^3^) | Single-pollutant  model | Dual-pollutant model 1 | Dual-pollutant model 2 | Dual-pollutant model 3 |
| --- | --- | --- | --- | --- |
| **Total** | | | | |
| PM_2.5_ | PM_2.5_ | +O_3_ | +SO_2_ | +NO_2_ |
| L | ref | ref | ref | ref |
| M | 1.228 (1.091, 1.381) | 1.228 (1.091, 1.381) | 1.231 (1.094, 1.385) | 1.245 (1.105, 1.403) |
| H | 1.004 (0.798, 1.262) | 1.012 (0.801, 1.277) | 1.023 (0.810, 1.293) | 1.059 (0.833 1.346) |
| SO_2_ | SO_2_ | +O_3_ | +NO_2_ | +PM_2.5_ |
| L | ref | ref | ref | ref |
| M | 1.025 (0.880, 1.195) | 1.020 (0.875, 1.190) | 1.022 (0.877, 1.191) | 1.025 (0.879, 1.195) |
| H | 0.987 (0.784, 1.243) | 0.976 (0.774, 1.230) | 0.970 (0.770, 1.222) | 0.985 (0.781, 1.241) |
| NO_2_ | NO_2_ | +O_3_ | +SO_2_ | +PM_2.5_ |
| L | ref | ref | ref | ref |
| M | 0.918 (0.804, 1.047) | 0.919 (0.806, 1.049) | 0.912 (0.799, 1.001) | 0.919 (0.805, 1.048) |
| H | 0.981 (0.806, 1.194) | 0.987 (0.810, 1.202) | 0.991 (0.814, 1.207) | 0.988 (0.809, 1.207) |
| O_3_ | O_3_ | +NO_2_ | +SO_2_ | +PM_2.5_ |
| L | ref | ref | ref | ref |
| M | 1.099 (0.952, 1.269) | 1.099 (0.953, 1.269) | 1.096 (0.950, 1.265) | 1.100 (0.953, 1.270) |
| H | 1.072 (0.876, 1.311) | 1.072 (0.876, 1.311) | 1.073 (0.878, 1.313) | 1.073 (0.877, 1.312) |
| CO | CO | +NO_2_ | +SO_2_ | +PM_2.5_ |
| L | ref | ref | ref | ref |
| M | 1.169 (1.028, 1.329) | 1.182 (1.037, 1.347) | 1.176 (1.033, 1.339) | 1.170 (1.027, 1.332) |
| H | 0.957 (0.793, 1.154) | 0.987 (0.810, 1.202) | 0.973 (0.803, 1.180) | 0.958 (0.790, 1.162) |
| **Male** | | | | |
| PM_2.5_ | PM_2.5_ | +O_3_ | +SO_2_ | +NO_2_ |
| L | ref | ref | ref | ref |
| M | 1.181 (1.023, 1.364) | 1.182 (1.023, 1.364) | 1.187 (1.028, 1.370) | 1.208 (1.044, 1.398) |
| H | 0.884 (0.667, 1.171) | 0.881 (0.661, 1.173) | 0.917 (0.688 1.222) | 0.960 (0.715, 1.290) |
| SO_2_ | SO_2_ | +O_3_ | +NO_2_ | +PM_2.5_ |
| L | ref | ref | ref | ref |
| M | 1.021 (0.846, 1.231) | 1.018 (0.844, 1.229) | 1.015 (0.842, 1.224) | 1.017 (0.843, 1.227) |
| H | 1.099 (0.829, 1.456) | 1.092 (0.822, 1.450) | 1.065 (0.804, 1.412) | 1.076 (0.811, 1.428) |
| NO_2_ | NO_2_ | +O_3_ | +SO_2_ | +PM_2.5_ |
| L | ref | ref | ref | ref |
| M | 0.868 (0.738, 1.020) | 0.869 (0.739, 1.021) | 0.859 (0.730, 1.010) | 0.872 (0.742, 1.026) |
| H | 0.904 (0.711, 1.149) | 0.907 (0.714, 1.154) | 0.919 (0.723, 1.168) | 0.936 (0.734, 1.194) |
| O_3_ | O_3_ | +NO_2_ | +SO_2_ | +PM_2.5_ |
| L | ref | ref | ref | ref |
| M | 0.990 (0.831, 1.179) | 0.990 (0.831, 1.178) | 0.985 (0.827, 1.173) | 0.997 (0.837, 1.188) |
| H | 0.957 (0.747, 1.226) | 0.957 (0.747, 1.225) | 0.961 (0.750, 1.231) | 0.965 (0.753, 1.237) |
| CO | CO | +NO_2_ | +SO_2_ | +PM_2.5_ |
| L | ref | ref | ref | ref |
| M | 1.117 (0.954, 1.308) | 1.141 (0.972, 1.340) | 1.131 (0.964, 1.327) | 1.134 (0.966, 1.332) |
| H | 0.900 (0.716, 1.133) | 0.956 (0.751, 1.217) | 0.933 (0.737, 1.181) | 0.930 (0.734, 1.178) |
| **Female** | | | | |
| PM_2.5_ | PM_2.5_ | +O_3_ | +SO_2_ | +NO_2_ |
| L | ref | ref | ref | ref |
| M | 1.338 (1.090, 1.643) | 1.337 (1.089, 1.642) | 1.336 (1.088, 1.641) | 1.337 (1.086, 1.646) |
| H | 1.303 (0.878, 1.935) | 1.340 (0.895, 2.004) | 1.287 (0.860, 1.925) | 1.299 (0.859, 1.965) |
| SO_2_ | SO_2_ | +O_3_ | +NO_2_ | +PM_2.5_ |
| L | ref | ref | ref | ref |
| M | 1.027 (0.786, 1.341) | 1.019 (0.779, 1.331) | 1.028 (0.787, 1.343) | 1.031 (0.790, 1.346) |
| H | 0.790 (0.530, 1.178) | 0.776 (0.520, 1.158) | 0.794 (0.531, 1.186) | 0.813 (0.544, 1.216) |
| NO_2_ | NO_2_ | +O_3_ | +SO_2_ | +PM_2.5_ |
| L | ref | ref | ref | ref |
| M | 1.027 (0.818, 1.289) | 1.031 (0.821, 1.294) | 1.030 (0.820, 1.294) | 1.022 (0.814, 1.284) |
| H | 1.150 (0.815, 1.622) | 1.162 (0.823, 1.640) | 1.145 (0.811, 1.618) | 1.098 (0.773, 1.561) |
| O_3_ | O_3_ | +NO_2_ | +SO_2_ | +PM_2.5_ |
| L | ref | ref | ref | ref |
| M | 1.364 (1.060, 1.755) | 1.364 (1.059, 1.755) | 1.368 (1.063, 1.762) | 1.350 (1.048, 1.738) |
| H | 1.351 (0.954, 1.912) | 1.350 (0.954, 1.911) | 1.349 (0.953, 1.909) | 1.337 (0.945, 1.893) |
| CO | CO | +NO_2_ | +SO_2_ | +PM_2.5_ |
| L | ref | ref | ref | ref |
| M | 1.285 (1.030, 1.604) | 1.274 (1.018, 1.594) | 1.277 (1.022, 1.596) | 1.249 (0.998, 1.563) |
| H | 1.089 (0.786, 1.509) | 1.063 (0.755, 1.497) | 1.071 (0.766, 1.497) | 1.029 (0.735. 1.440) |
| **<60 years** | | | | |
| PM_2.5_ | PM_2.5_ | +O_3_ | +SO_2_ | +NO_2_ |
| L | ref | ref | ref | ref |
| M | 1.223 (1.065, 1.403) | 1.222 (1.064, 1.402) | 1.229 (1.071, 1.411) | 1.237 (1.076, 1.423) |
| H | 1.065 (0.813, 1.395) | 1.085 (0.825, 1.429) | 1.107 (0.840, 1.459) | 1.111 (0.837, 1.476) |
| SO_2_ | SO_2_ | +O_3_ | +NO_2_ | +PM_2.5_ |
| L | ref | ref | ref | ref |
| M | 1.026 (0.856, 1.230) | 1.018 (0.849, 1.222) | 1.024 (0.854, 1.227) | 1.027 (0.857, 1.232) |
| H | 0.955 (0.728, 1.251) | 0.939 (0.716, 1.231) | 0.942 (0.718, 1.236) | 0.960 (0.731, 1.260) |
| NO_2_ | NO_2_ | +O_3_ | +SO_2_ | +PM_2.5_ |
| L | ref | ref | ref | ref |
| M | 0.979 (0.838, 1.143) | 0.981 (0.840, 1.146) | 0.970 (0.831, 1.133) | 0.977 (0.837, 1.142) |
| H | 1.008 (0.797, 1.275) | 1.017 (0.804, 1.287) | 1.024 (0.809, 1.295) | 1.001 (0.787, 1.271) |
| O_3_ | O_3_ | +NO_2_ | +SO_2_ | +PM_2.5_ |
| L | ref | ref | ref | ref |
| M | 1.133 (0.956, 1.342) | 1.133 (0.957, 1.342) | 1.127 (0.951, 1.335) | 1.131 (0.954, 1.340) |
| H | 1.180 (0.931, 1.497) | 1.181 (0.931, 1.497) | 1.184 (0.933, 1.501) | 1.178 (0.929, 1.495) |
| CO | CO | +NO_2_ | +SO_2_ | +PM_2.5_ |
| L | ref | ref | ref | ref |
| M | 1.288 (1.109, 1.497) | 1.302 (1.117, 1.517) | 1.308 (1.123, 1.522) | 1.285 (1.103, 1.497) |
| H | 1.066 (0.852, 1.333) | 1.097 (0.866, 1.388) | 1.109 (0.881, 1.397) | 1.060 (0.842 1.334) |
| **≥60 years** | | | | |
| PM_2.5_ | PM_2.5_ | +O_3_ | +SO_2_ | +NO_2_ |
| L | ref | ref | ref | ref |
| M | 1.236 (0.985, 1.552) | 1.235 (0.984, 1.551) | 1.233 (0.982, 1.548) | 1.259 (0.999, 1.584) |
| H | 0.866 (0.562, 1.334) | 0.843 (0.542, 1.311) | 0.843 (0.542, 1.310) | 0.932 (0.593, 1.465) |
| SO_2_ | SO_2_ | +O_3_ | +NO_2_ | +PM_2.5_ |
| L | ref | ref | ref | ref |
| M | 1.022 (0.768, 1.362) | 1.025 (0.768, 1.366) | 1.017 (0.764, 1.355) | 1.020 (0.765, 1.359) |
| H | 1.082 (0.697, 1.679) | 1.087 (0.698, 1.694) | 1.052 (0.679, 1.632) | 1.063 (0.684, 1.653) |
| NO_2_ | NO_2_ | +O_3_ | +SO_2_ | +PM_2.5_ |
| L | ref | ref | ref | ref |
| M | 0.772 (0.601, 0.991) | 0.772 (0.601, 0.991) | 0.771 (0.600, 0.991) | 0.776 (0.604, 0.996) |
| H | 0.903 (0.629, 1.296) | 0.902 (0.628, 1.296) | 0.904 (0.629, 1.298) | 0.943 (0.653, 1.362) |
| O_3_ | O_3_ | +NO_2_ | +SO_2_ | +PM_2.5_ |
| L | ref | ref | ref | ref |
| M | 1.025 (0.782, 1.343) | 1.025 (0.783, 1.342) | 1.026 (0.783, 1.345) | 1.033 (0.788, 1.354) |
| H | 0.843 (0.577, 1.232) | 0.842 (0.576, 1.230) | 0.842 (0.576, 1.230) | 0.848 (0.580, 1.241) |
| CO | CO | +NO_2_ | +SO_2_ | +PM_2.5_ |
| L | ref | ref | ref | ref |
| M | 0.898 (0.701, 1.152) | 0.910 (0.708, 1.170) | 0.890 (0.694, 1.142) | 0.907 (0.705, 1.166) |
| H | 0.721 (0.510, 1.020) | 0.750 (0.523, 1.076) | 0.701 (0.491, 1.001) | 0.735 (0.515, 1.049) |
| **Smear positive** | | | | |
| PM_2.5_ | PM_2.5_ | +O_3_ | +SO_2_ | +NO_2_ |
| L | ref |  |  |  |
| M | 1.230 (0.969, 1.562) | 1.233 (0.971, 1.564) | 1.233 (0.971, 1.566) | 1.226 (0.962, 1.562) |
| H | 0.962 (0.603, 1.534) | 0.914 (0.568, 1.469) | 0.981 (0.608, 1.582) | 0.949 (0.581, 1.553) |
| SO_2_ | SO_2_ | +O_3_ | +NO_2_ | +PM_2.5_ |
| L | ref | ref | ref | ref |
| M | 1.281 (0.944, 1.739) | 1.295 (0.954, 1.760) | 1.283 (0.945, 1.743) | 1.281 (0.944, 1.739) |
| H | 1.334 (0.839, 2.121) | 1.366 (0.856, 2.182) | 1.345 (0.841, 2.151) | 1.334 (0.835, 2.130) |
| NO_2_ | NO_2_ | +O_3_ | +SO_2_ | +PM_2.5_ |
| L | ref | ref | ref | ref |
| M | 0.973 (0.746, 1.269) | 0.969 (0.743, 1.265) | 0.964 (0.739, 1.258) | 0.976 (0.748, 1.273) |
| H | 1.273 (0.848, 1.911) | 1.258 (0.837, 1.892) | 1.287 (0.857, 1.932) | 1.299 (0.860, 1.962) |
| O_3_ | O_3_ | +NO_2_ | +SO_2_ | +PM_2.5_ |
| L | ref | ref | ref | ref |
| M | 0.922 (0.688, 1.236) | 0.922 (0.687, 1.236) | 0.920 (0.686, 1.234) | 0.923 (0.688, 1.238) |
| H | 0.917 (0.613, 1.371) | 0.916 (0.613, 1.371) | 0.920 (0.615, 1.376) | 0.918 (0.613, 1.373) |
| CO | CO | +NO_2_ | +SO_2_ | +PM_2.5_ |
| L | ref | ref | ref | ref |
| M | 0.938 (0.725, 1.213) | 0.935 (0.721, 1.212) | 0.946 (0.730, 1.226) | 0.939 (0.723, 1.219) |
| H | 0.954 (0.652, 1.397) | 0.944 (0.634, 1.407) | 0.975 (0.660, 1.440) | 0.956 (0.647, 1.413) |
| **Smear negative** | | | | |
| PM_2.5_ | PM_2.5_ | +O_3_ | +SO_2_ | +NO_2_ |
| L | ref | ref | ref | ref |
| M | 1.225 (1.068, 1.404) | 1.224 (1.068, 1.404) | 1.228 (1.071, 1.408) | 1.249 (1.087, 1.434) |
| H | 1.018 (0.781, 1.326) | 1.048 (0.800, 1.372) | 1.039 (0.793, 1.361) | 1.095 (0.829, 1.444) |
| SO_2_ | SO_2_ | +O_3_ | +NO_2_ | +PM_2.5_ |
| L | ref | ref | ref | ref |
| M | 0.951 (0.795, 1.137) | 0.940 (0.785, 1.125) | 0.947 (0.792, 1.133) | 0.950 (0.794, 1.137) |
| H | 0.877 (0.671, 1.146) | 0.854 (0.653, 1.117) | 0.859 (0.657, 1.122) | 0.875 (0.669, 1.145) |
| NO_2_ | NO_2_ | +O_3_ | +SO_2_ | +PM_2.5_ |
| L | ref | ref | ref | ref |
| M | 0.900 (0.772, 1.049) | 0.903 (0.775, 1.052) | 0.895 (0.767, 1.043) | 0.900 (0.772, 1.050) |
| H | 0.904 (0.721, 1.135) | 0.914 (0.728, 1.147) | 0.914 (0.728, 1.147) | 0.906 (0.719, 1.142) |
| O_3_ | O_3_ | +NO_2_ | +SO_2_ | +PM_2.5_ |
| L | ref | ref | ref | ref |
| M | 1.195 (1.012, 1.411) | 1.194 (1.012, 1.410) | 1.191 (1.009, 1.407) | 1.196 (1.013, 1.413) |
| H | 1.161 (0.917, 1.468) | 1.160 (0.917, 1.467) | 1.162 (0.919, 1.470) | 1.162 (0.918, 1.470) |
| CO | CO | +NO_2_ | +SO_2_ | +PM_2.5_ |
| L | ref | ref | ref | ref |
| M | 1.261 (1.086, 1.466) | 1.282 (1.101, 1.493) | 1.269 (1.091, 1.476) | 1.262 (1.083, 1.469) |
| H | 0.968 (0.778, 1.203) | 1.013 (0.806, 1.273) | 0.984 (0.787, 1.231) | 0.968 (0.773, 1.211) |

PM_2.5_: L = ≤86 ug/m^3^, M = (87-122) ug/m^3^, H = ＞123ug/m^3^;

SO_2_: L = ≤58 ug/m^3^, M = (59-97) ug/m^3^, H = ＞98ug/m^3^;

NO_2_: L = ≤50 ug/m^3^, M = (51-65) ug/m^3^, H= ＞66ug/m^3^;

O_3_: L = ≤88 ug/m^3^, M = (89-141) ug/m^3^, H = ＞142 ug/m^3^;

CO: L = ≤1228 ug/m^3^, M = (1229-1638) ug/m^3^, H = ＞1639ug/m^3^.
